# Intake of fibre and plant foods and the risk of abdominal aortic aneurysm in a large prospective cohort study in Sweden

**DOI:** 10.1007/s00394-019-02054-w

**Published:** 2019-07-22

**Authors:** Sara Bergwall, Stefan Acosta, Emily Sonestedt

**Affiliations:** 1 Vascular Diseases, Department of Clinical Sciences Malmö, Lund University, Ruth Lundskogs gata 10, SE-214 28 Malmö, Sweden; 2 Vascular Centre, Department of Cardiothoracic and Vascular Surgery, Skåne University Hospital, Ruth Lundskogs gata 10, SE-214 28 Malmö, Sweden; 3 Nutritional Epidemiology, Department of Clinical Sciences Malmö, Lund University, Jan Waldenströms gata 35, SE-214 28 Malmö, Sweden

**Keywords:** Fibre, Abdominal aortic aneurysm, Diet, Fruits, Vegetables, Prospective study

## Abstract

**Purpose:**

The purpose of this study was to investigate fibre, and plant foods, and its association with AAA risk.

**Methods:**

In this prospective cohort study, Malmö Diet and Cancer Study, baseline data collection was carried out 1991–1996. The study participants’ (*n* = 26,133) dietary habits were extensively recorded at baseline. The specific diagnosis of AAA in the in-hospital registry was found valid in 95%. The association between plant foods, such as cereals and types of vegetables, and AAA was assessed by using Cox regression analysis expressed as hazard ratios (HR) with 95% confidence intervals (CI).

**Results:**

A high intake of fibre was independently associated with AAA risk (HR per quintile 0.87, 95% CI 0.79–0.97). High intake of vegetables (HR 0.91, 95% CI 0.84–0.98), specifically leaf vegetables (HR 0.87, 95% CI 0.81–0.94), and fruits and berries (HR 0.89, 95% CI 0.82–0.96), citrus (HR 0.91, 95% CI 0.85–0.98) and non-citrus fruits (HR 0.87, 95% CI 0.81–0.95) were independently associated with a decreased AAA risk.

**Conclusions:**

A high intake of fruits and berries and vegetables, in particular leaf vegetables, are associated with a decreased risk of developing AAA.

**Electronic supplementary material:**

The online version of this article (10.1007/s00394-019-02054-w) contains supplementary material, which is available to authorized users.

## Background

Abdominal aortic aneurysm (AAA) is a disease leading to a dilation of the aorta, which can have fatal outcomes if rupture occurs. An AAA is located between the diaphragm and the aortic bifurcation and is generally defined as an aortic diameter exceeding 30 mm [1].

AAA has a complex, not yet fully deciphered, pathogenesis. However, a number of risk factors for AAA has been identified and thoroughly studied. The non-modifiable risk factors for AAA are primarily advanced age, male sex and heredity [1]. Smoking is the main recognized and established modifiable risk factor for AAA, with almost five times higher risk among current versus never smokers [2]. Excessive alcohol consumption has also been suggested as a risk factor for AAA and consuming less than two units of alcohol per day may lead to a risk reduction [3]. Furthermore, a meta-analysis identified that individuals with coronary artery disease (CAD) have a higher risk of developing AAA in the future, making CAD a solid predictor of AAA [4]. However, a negative association between diabetes mellitus and AAA has been identified [5, 6].

The global incidence of AAA seems to be decreasing, primarily due to a reduction in number of smokers. However, it is highly plausible that the prevalence and incidence of AAA is higher than estimated, as many sudden deaths are not diagnosed due to post-mortem examinations not being conducted [1]. In 2013, a meta-analysis was conducted on AAA prevalence in the global population. This study found that the pooled prevalence, from the 56 included articles, was 6.0% for men and 1.6% for women. The 65–74-year age group had the highest AAA prevalence. However, the authors emphasized that there are major regional differences in AAA prevalence, in part due to variances in age and gender distributions and the use of different definitions for AAA [7]. In Sweden, AAA prevalence is estimated to be approximately 1.5% amongst men aged above 65 years who participates in screening. In addition, 400–500 deaths in men ≥ 65 years are ascribed to ruptured AAAs (rAAA) annually in Sweden [8].

Maintaining a high-quality diet, a potential protector against a plethora of diseases [9–11], has been scarcely investigated in association with AAA risk. In the Malmö Diet and Cancer cohort, we did not find significant association between adherence to a high-quality diet, as defined by a diet quality index, and AAA risk. The diet quality index consist of six dietary components and an association between high intake of fruits and vegetables and reduced AAA risk was shown [12], as well as a trend towards an association between adherence to recommendations for fibre and AAA risk. [12]. To fully comprehend the importance of diet, we need to study both entire diet patterns and isolated dietary components. A retrospective cross-sectional cohort study in the US has identified an association between fruit and vegetable intake and AAA risk [6], and a Swedish prospective cohort study found an association between fruit, but not vegetable, intake and risk of developing AAA [13]. However, the evidence for the importance of fibre and plant foods, including cereals and types of vegetables, on AAA risk are scarce. The results from our previous study in the MDC, combined with the paucity of research on the topic, indicates that we need to go further into detail and focus explicitly on fibre and plant foods and the association with AAA.

The European Food Safety Association (EFSA) defines fibre as “non-digestible carbohydrates plus lignin” [14]. Fibre is found in for instance fruits, vegetables and cereals. A high intake of fibre has been shown to be associated with a reduced risk of diseases, such as colorectal cancer [15] and diabetes mellitus [16]. Adhering to the dietary recommendations for fibre has also been shown to be associated with maintaining normal blood cholesterol and blood glucose levels [17]. While cholesterol [18] is associated with AAA risk, glucose [5, 19] is inversely associated with AAA, and the net effect is unknown.

Due to the scarce research investigating dietary components and AAA, and the potential health benefits associated with a high intake of fibre, the aim of the present study was to investigate the association between fibre and plant foods and the risk of developing AAA.

## Methods

### Study population and data collection

The Malmö Diet and Cancer Study (MDCS) is a prospective cohort study, where baseline data collection occurred between 1991 and 1996. The cohort comprises 30,446 individuals who were born between 1923 and 1950. In addition to the age requirement, the participants had to reside in Malmö, Sweden, and be proficient in Swedish to be considered eligible to participate in the study [20]. Individuals who had complete dietary assessment, anthropometric measurements and had filled in a lifestyle questionnaire were included in the study (*n* = 28,098). Out of the 28,098 individuals, those with prevalent AAA (*n* = 24), cardiovascular disease, defined as myocardial infarction or stroke (*n* = 818), or diabetes mellitus (*n* = 1230) were excluded. This resulted in a study population of 26,133. At baseline, all study participants gave written informed consent and the study has ethical clearance from the Regional Ethical Review Board in Lund, Sweden (Dnr §LU5190).

### Diet variables

Dietary habits were collected at baseline through a combination of a 7-day food diary, a 168-item food frequency questionnaire and a 1-h interview [21]. The food diary recorded food intake at prepared meals (usually lunch and dinner meals) as well as intake of cold beverages and nutrient supplements. The food frequency questionnaire collected information on intake of other foods than prepared meals during the preceding year. A picture booklet was also included in the questionnaire to evaluate portion sizes. The 1-h interview made sure that the reported food consumption did not overlap. Detailed information on cooking practises, portion sizes and recipes of the foods recorded in the diary was also gathered during the interview [21].

Average daily food intake (g/day) was calculated by combining the information from the food diary and the questionnaire. Intake of bread and cereals were collected in the questionnaire and were thereafter categorized based on the fibre content into: low-fibre soft bread (< 5.9% fibre), high-fibre soft bread (> 6% fibre), low-fibre crisp bread (< 10% fibre), high-fibre crisp bread and rusks (> 10% fibre), high-fibre cereals (> 10% fibre) and low-fibre cereals (< 10% fibre). The summary variable whole grains (servings/day) include all high-fibre bread and cereals and the summary variable refined grains (servings/day) contain all low-fibre bread and cereals. Fruits were divided into citrus and non-citrus fruits. Vegetables were, in addition to total vegetables, divided into root vegetables (including carrots), leaf vegetables (including kale), carrots and cabbage. In 1991, information was not included in the database on leaf vegetables, cabbages and root vegetables, resulting in missing data for 2010 participants. Juice included all types of vegetable and citrus juices.

Total energy intake (kcal/day, including alcohol and fibre), and fibre intake (g/day) was estimated by combining intakes from foods and supplements with the food composition database. Fibre intake expressed as the percentage of total energy intake (expressed as E%) was calculated. The participants were divided into quintiles based on dietary intake. For the dietary variables with more than 25% zero-consumers (i.e. wholegrain cereals, refined cereals, rice/pasta, biscuits and rusks, high-fibre soft bread, high-fibre crisp bread, cabbage and juice), the zero-consumers were placed in one group and the remainder of the participants were divided into tertiles.

### Other variables

To attain the variables age and sex, the civic registration number of each participant was used. At baseline, nurses conducted measurements on height, weight and blood pressure after 10 min of rest. Body mass index (BMI) was calculated using weight and height and the formula kg/m^2^, and was expressed as kg per m^2^. Information on BMI was missing for 31 individuals. Hypertension was defined as a systolic blood pressure ≥ 130 mm Hg or diastolic blood pressure ≥ 85 mmHg DBP, or if the individual used antihypertensive medication.

At baseline, a questionnaire was used to attain data on the lifestyle of the study participants. Smoking status, where data on 10 individuals was missing, was divided into three categories: never smoker, previous smoker and current smoker. In addition, number of cigarettes per day was calculated for the smokers and divided into quintiles, resulting in the following divisions: ≤ 7, 8–10, 11–15, 16–20 and ≥ 21. Information was missing for 429 individuals for number of cigarettes per day. Individuals who reported that they did not smoke any cigarettes were classified as zero-consumers.

For education, five groups, based on the highest level of education attained were established as follows: less than 9 years, elementary school (9–10 years), upper secondary school (11–13 years), university without a degree and university degree. Information on education status was missing for 915 individuals. Alcohol consumption was divided into gender-specific quintiles based on the current consumption of the participants, as reported in the 7-day food diary. Individuals who reported no alcohol consumption the previous year in the lifestyle questionnaire and no alcohol in the 7-day diary were categorized as zero-consumers. Leisure-time physical activity was estimated based on the duration of 17 activities and expressed as Metabolic Equivalent of Task (MET) hours per week. This variable was divided into five groups with the following divisions expressed as MET-h/week: 0–7.5, 7.5–15, 15–25, 25–50 and > 50. Physical activity was missing for 175 individuals in the study. The variables, that were included in the models, were chosen because of being potential confounders, i.e. being associated with dietary intake and being suspected risk factor for AAA. We have chosen to not include intermediary factors such as hypertension in the statistical models. However, we have run an additional model with hypertension in the model.

There was a change in the coding routine in 1994 [22], where the dietary interview was shortened from 60 to 45 min, and hence the variable ‘method’ was created. Season refers to the time of year that the data collection occurred. Misreporters were identified by comparing the individually reported intake with their total energy expenditure. A 95% confidence interval (CI) for total energy expenditure was set and individuals who had reported energy intake above or below the CI were classified as misreporters. The questionnaire question ‘have you substantially changed your dietary habits in the past?’ was used to identify dietary changers [23].

### Endpoint ascertainment

By linking the civic registration number of each included participant in the MDCS with registers, information on first diagnosis of AAA, ruptured AAA or surgical procedure for AAA were attained. The registers used for this study were the Cause of Death Register, where all deaths in Sweden are registered as well as the cause of death as noted in the death certificate, and the Inpatient and Outpatient Register, where all hospitalisations in Sweden are registered as well as procedural and surgical codes. The International Classification of Disease (ICD) versions 8, 9 and 10 were used for both registers. The corresponding ICD 10 codes were 171.3 for rAAA and 171.4 for AAA. The latest point of follow-up for this study was 31 December 2016.

Validation of the AAA diagnosis was conducted on individuals diagnosed with AAA during 2016. In all, 173 patients were diagnosed with AAA or rAAA between January 1st and December 31st, 2016. The proportion of individuals with ruptured and intact AAA was 18% and 82%, respectively. Therefore, 82 patients with diagnosis of AAA (I71.4) and 18 patients with ruptured AAA (I71.3) were randomly selected for the validation procedure using patient record data. Two rAAA patients were simultaneously diagnosed with AAA and, therefore, 98 patients remained for validation. Differences in characteristics between patients with AAA and rAAA are shown in supplementary Table 3. The diagnosis of AAA or rAAA was confirmed in 94.9% (93/98) of the patients. The five misdiagnoses were: thoracic aortic aneurysm (*n* = 1), common iliac aneurysm (*n* = 1), multiple mycotic pseudoaneurysm in the abdominal aorta (*n* = 1), chronic type B aortic dissection with secondary thoraco-abdominal aneurysm formation (*n* = 1) and lower extremity artery stenosis (*n* = 1).

### Statistical analyses

The analyses were carried out in IBM SPSS, version 25 (SPSS Inc, Chicago, IL). The chosen level of statistical significance was set at 0.05. The baseline characteristics of the study participants were expressed as median (IQR), mean (SD) or count (%). We calculated hazard ratios (HR) using the Cox proportional hazards regression model with years of follow-up as the time scale. HR per 1 SD increment was calculated for age, BMI and dietary variables. The HR for the included lifestyle factors were adjusted for age and sex and the HR for the diet variables were adjusted for age, sex, diet assessment method and total energy intake. For the main analyses, we calculated HR for the dietary components divided in quintiles (or zero-consumers plus tertiles). The basic model included adjustments for age, sex, method and total energy intake. In the multivariable model adjustments for physical activity, education, alcohol, smoking and BMI were added. In a sensitivity analyses, misreporters and dietary changers were excluded, with same adjustments as in the multivariable model.

In post hoc analyses, we run the analyses for the key findings separately in smokers (i.e. regularly or occasionally smokers) and non-smokers (i.e. former and never smokers). We examined if smoking status modified the association by including an interaction term, consisting of a cross product of smoking status and the quintiles or tertiles of the dietary variables, in addition to the smoking, diet and confounding variables in the model.

## Results

### Demographic baseline characteristics

This study consisted of 26,133 participants, with 429 incident AAA individuals and a cumulative AAA incidence of 1.7%. Out of the 429 individuals with AAA, 63 were diagnosed with rAAA. The mean follow-up time for the participants was 20.7 years. Table 1 outlines the baseline characteristics of the study participants. Individuals who developed AAA were more often males and smokers, and consumed less fibre, fruits and vegetables, compared to non-AAA individuals. Participants with AAA also maintained a lower degree of leisure-time physical activity.

**Table 1:** Baseline characteristics according to incident AAA cases at follow-up on December 31, 2016 in the Malmö Diet and Cancer Cohort

Variables	Incident AAA cases (*n* = 429)	Non-AAA cases (*n* = 25,704)	HR (95% CI)
Males (%)	315 (73.4)	9561 (37.2)	4.75 (3.83*–*5.90)
Age (years)	60.74 (8.82)	57.80 (7.60)	1.75 (1.57*–*1.94)
BMI (kg/m^2^)	25.60 (3.91)	26.18 (3.80)	1.07 (0.96*–*1.18)
Fibre (E%)	1.63 (0.56)	1.81 (0.54)	0.71 (0.63*–*0.80)
Vegetables (g/1000 kcal)	59.53 (48.20)	74.50 (56.37)	0.74 (0.65*–*0.85)
Fruits and berries (g/1000 kcal)	52.26 (66.68)	79.22 (75.72)	0.70 (0.61*–*0.80)
Juice (g/1000 kcal)	0.03 (25.92)	0.31 (45.07)	0.94 (0.83*–*1.05)
Whole grains (servings/1000 kcal)	0.11 (0.25)	0.15 (0.26)	0.93 (0.83*–*1.04)
Refined grains (servings/1000 kcal)	1.10 (0.68)	1.07 (0.64)	0.99 (0.90*–*1.09)
Potatoes (g/1000 kcal)	58.25 (34.72)	49.59 (35.30)	1.18 (1.08*–*1.28)
Smoking status			
Never	49 (11.4)	10,027 (39.0)	1.00
Current	256 (59.7)	7181 (27.9)	9.21 (6.76*–*12.55)
Former	124 (28.9)	8466 (33.0)	2.44 (1.75*–*3.41)
Cigarettes per day			
Zero-consumption	204 (47.6)	19,230 (74.8)	1.00
Quintile 1	24 (5.6)	1452 (5.6)	2.14 (1.40*–*3.30)
Quintile 2	39 (9.1)	1278 (5.0)	4.59 (3.26*–*6.48)
Quintile 3	64 (14.9)	1628 (6.3)	6.34 (4.78*–*8.42)
Quintile 4	72 (16.8	1527 (5.9)	6.88 (5.22*–*9.05)
Quintile 5	26 (6.1)	589 (2.3)	5.80 (3.83*–*8.80)
Alcohol consumption			
Zero-consumption	27 (6.3)	1570 (6.1)	1.00
Quintile 1	83 (19.3)	4715 (18.3)	0.82 (0.53*–*1.27)
Quintile 2	77 (17.9)	4760 (18.5)	0.71 (0.46*–*1.10)
Quintile 3	90 (21.0)	4867 (18.9)	0.82 (0.53*–*1.27)
Quintile 4	71 (16.6)	4883 (19.0)	0.67 (0.43*–*1.04)
Quintile 5	81 (18.9)	4909 (19.1)	0.87 (0.56*–*1.35)
Education			
Less than 9 years	233 (54.3)	10,500 (40.8)	1.00
Elementary school (9–10 years)	89 (20.7)	6815 (26.5)	0.77 (0.60*–*0.98)
Upper secondary school (11*–*13 years)	31 (7.2)	2297 (8.9)	0.58 (0.40*–*0.84)
University without a degree	35 (8.2)	2266 (8.8)	0.76 (0.53*–*1.08)
University degree	40 (9.3)	3768 (14.7)	0.58 (0.42*–*0.82)
Leisure-time physical activity			
0*–*7.5 MET-h/week	55 (12.8)	2390 (9.3)	1.00
7.5*–*15 MET-h/week	80 (18.6)	3799 (14.8)	0.89 (0.63*–*1.25)
15*–*25 MET-h/week	88 (20.5)	5874 (22.9)	0.62 (0.44*–*0.87)
25*–*50 MET-h/week	142 (33.1)	9375 (36.5)	0.60 (0.44*–*0.81)
> 50 MET-h/week	60 (14.0)	4091 (15.9)	0.50 (0.35*–*0.73)
Hypertension	381 (88.8)	19,583 (76.2)	1.72 (1.27*–*2.34)

Data are expressed as: mean (SD), median (IQR) or *n* (%) for cases and non-cases. Diet variables are adjusted for age, sex, total energy intake, diet assessment method and season and expressed as median (IQR). HR per 1 SD increment was calculated for age, BMI and dietary intakes. HR for lifestyle factors were adjusted for age and sex

### The association between intake of fibres, fruits and vegetables and AAA risk

In the basic model, linear protective associations were observed between intake of fibre, whole grains, total vegetables, leaf vegetables, carrots, total fruits and berries, citrus fruits and non-citrus fruits and AAA risk, while intake of potato showed a positive association with AAA risk (Table 2). No association was observed for refined grains or root vegetables. After adjusting for numerous potential confounders, the linear associations were attenuated, but remained significant for total vegetables, leaf vegetables, total fruits, citrus fruits and non-citrus fruits (negative associations) as well as for potato (positive association). For example, individuals in the highest intake group of leaf vegetables (24 g/1000 kcal on average) has a 47% decreased risk compared to individuals in the lowest intake group (0 g on average). After excluding misreporters and dietary changers in the sensitivity analysis, the associations remained virtually unchanged; however, the association with total vegetables were strengthened, and the associations with fibre and whole grains became statistically significant. The group with the highest fibre intake (2.6 E% on average) had 38% decreased risk of AAA compared to the group with the lowest intake (1.2 E% on average). The highest quintile of vegetables consumers had an HR of 0.57 (95% CI 0.35–0.93) when compared to the lowest quintile in the sensitivity analysis.

**Table 2:** HR and 95% CI for energy-adjusted food groups, divided according to categories of intake among participants in the Malmö Diet and Cancer Cohort

	Intake categories					
	1	2	3	4	5	Per intake category
Fiber						
Cases/follow-up	147/19.1	89/19.7	81/20.1	55/20.4	57/20.6	
Mean intake (E%)	1.16	1.50	1.74	2.03	2.64	
Basic model^a^	1.00	0.61 (0.47*–*0.80)	0.56 (0.42*–*0.74)	0.41 (0.30*–*0.56)	0.46 (0.33*–*0.63)	0.80 (0.74*–*0.86)
Multivariable model^b^	1.00	0.74 (0.57*–*0.98)	0.82 (0.62*–*1.09)	0.64 (0.46*–*0.89)	0.80 (0.57*–*1.12)	0.93 (0.86*–*1.00)
Excluding misreporters^c^	1.00	0.76 (0.56*–*1.04)	0.68 (0.47*–*0.97)	0.63 (0.43*–*0.94)	0.62 (0.38*–*1.00)	0.87 (0.79*–*0.97)
Whole grains						
Cases/follow-up	121/19.2	93/20.0	76/20.2	74/20.2	65/20.5	
Mean intake (servings/1000 kcal)	0.01	0.07	0.16	0.28	0.59	
Basic model^a^	1.00	0.79 (0.60*–*1.04)	0.66 (0.50*–*0.88)	0.65 (0.48*–*0.86)	0.60 (0.44*–*0.81)	0.88 (0.82*–*0.94)
Multivariable model^b^	1.00	0.92 (0.70*–*1.21)	0.83 (0.62*–*1.11)	0.84 (0.63*–*1.14)	0.79 (0.58*–*1.09)	0.94 (0.88*–*1.01)
Excluding misreporters^c^	1.00	1.03 (0.75*–*1.43)	0.84 (0.59*–*1.19)	0.85 (0.59*–*1.22)	0.64 (0.41*–*1.00)	0.91 (0.83*–*0.99)
Refined grains						
Cases/follow-up	84/20.1	64/20.0	101/19.9	80/20.0	100/20.0	
Mean intake (servings/1000 kcal)	0.50	0.84	1.08	1.34	1.88	
Basic model^a^	1.00	0.75 (0.54*–*1.03)	1.11 (0.83*–*1.50)	0.85 (0.62*–*1.15)	0.95 (0.71*–*1.28)	1.00 (0.94*–*1.07)
Multivariable model^b^	1.00	0.74 (0.53*–*1.03)	1.19 (0.89*–*1.60)	0.89 (0.65*–*1.22)	1.07 (0.79*–*1.45)	1.03 (0.96*–*1.11)
Excluding misreporters^c^	1.00	0.80 (0.53*–*1.20)	1.18 (0.81*–*1.71)	0.91 (0.62*–*1.35)	1.05 (0.71*–*1.55)	1.02 (0.94*–*1.12)
Potatoes						
Cases/follow-up	48/20.4	77/20.2	85/20.0	106/19.8	113/19.6	
Mean intake (g/1000 kcal)	19	37	50	65	97	
Basic model^a^	1.00	1.44 (1.00*–*2.06)	1.48 (1.04*–*2.11)	1.71 (1.22*–*2.42)	1.72 (1.22*–*2.41)	1.12 (1.04*–*1.20)
Multivariable model^b^	1.00	1.43 (0.99*–*2.05)	1.46 (1.02*–*2.09)	1.56 (1.11*–*2.21)	1.53 (1.08*–*2.16)	1.08 (1.01*–*1.16)
Excluding misreporters^c^	1.00	1.11 (0.70*–*1.76)	1.63 (1.07*–*2.48)	1.36 (0.89*–*2.08)	1.32 (0.86*–*2.03)	1.06 (0.97*–*1.16)
Total vegetables						
Cases/follow-up	144/18.9	106/19.8	67/20.2	61/20.4	51/20.8	
Mean intake (g/1000 kcal)	31	55	74	99	163	
Basic model^a^	1.00	0.77 (0.60*–*0.99)	0.54 (0.40*–*0.73)	0.57 (0.42*–*0.77)	0.53 (0.38*–*0.75)	0.84 (0.78*–*0.90)
Multivariable model^b^	1.00	0.85 (0.66*–*1.10)	0.67 (0.50*–*0.91)	0.75 (0.55*–*1.03)	0.71 (0.51*–*1.01)	0.91 (0.84*–*0.98)
Excluding misreporters^c^	1.00	0.82 (0.61*–*1.11)	0.57 (0.40*–*0.83)	0.62 (0.41*–*0.93)	0.57 (0.35*–*0.93)	0.85 (0.77*–*0.94)
Leaf vegetables						
Cases/follow-up	121/18.6	100/19.6	77/20.1	56/20.3	35/20.4	
Mean intake (g/1000 kcal)	0	2	5	10	24	
Basic model^a^	1.00	0.97 (0.70*–*1.35)	0.82 (0.58*–*1.16)	0.52 (0.34*–*0.79)	0.45 (0.28*–*0.73)	0.81 (0.74*–*0.90)
Multivariable model^b^	1.00	0.93 (0.71*–*1.21)	0.93 (0.69*–*1.25)	0.70 (0.51*–*0.98)	0.53 (0.53*–*0.78)	0.87 (0.81*–*0.94)
Excluding misreporters^c^	1.00	1.04 (0.75*–*1.45)	1.06 (0.74*–*1.52)	0.63 (0.41*–*0.97)	0.62 (0.38*–*1.01)	0.88 (0.80*–*0.97)
Root vegetables						
Cases/follow-up	100/19.6	76/19.7	69/19.7	82/19.9	62/20.1	
Mean intake (g/1000 kcal)	1	5	10	17	40	
Basic model^a^	1.00	0.77 (0.57*–*1.04)	0.71 (0.52*–*0.96)	0.88 (0.66*–*1.19)	0.72 (0.52*–*0.996)	0.95 (0.88*–*1.02)
Multivariable model^b^	1.00	0.82 (0.61*–*1.12)	0.84 (0.62*–*1.15)	1.05 (0.78*–*1.41)	0.94 (0.68*–*1.31)	1.01 (0.94*–*1.09)
Excluding misreporters^c^	1.00	0.90 (0.62*–*1.29)	0.87 (0.59*–*1.27)	1.10 (0.76*–*1.59)	0.95 (0.62*–*1.44)	1.01 (0.92*–*1.11)
Carrots						
Cases/follow-up	102/19.5	88/19.6	70/19.8	71/20.0	58/20.1	
Mean intake (g/1000 kcal)	7	7	8	8	11	
Basic model^a^	1.00	0.88 (0.66*–*1.17)	0.73 (0.54*–*0.98)	0.79 (0.58*–*1.07)	0.70 (0.51*–*0.98)	0.92 (0.85*–*0.99)
Multivariable model^b^	1.00	0.95 (0.71*–*1.28)	0.84 (0.62*–*1.14)	0.96 (0.70*–*1.30)	0.94 (0.68*–*1.32)	0.98 (0.91*–*1.06)
Excluding misreporters^c^	1.00	1.02 (0.71*–*1.45)	1.00 (0.70*–*1.44)	1.03 (0.71*–*1.51)	0.95 (0.61*–*1.46)	0.99 (0.91*–*1.10)
Cabbage						
Cases/follow-up	239/19.96	64/20.21	63/19.95	63/19.96		
Mean intake (g/1000 kcal)	0	3	9	25		
Basic model^a^	1.00	0.90 (0.69*–*1.19)	0.88 (0.67*–*0.17)	0.93 (0.71*–*1.24)		0.97 (0.89*–*1.05)
Multivariable model^b^	1.00	0.93 (0.70*–*1.24)	0.91 (0.68*–*1.20)	0.98 (0.74*–*1.30)		0.98 (0.90*–*1.07)
Excluding misreporters^c^	1.00	1.00 (0.72*–*1.40)	0.86 (0.60*–*1.21)	0.99 (0.69*–*1.42)		0.98 (0.88*–*1.09)
All fruits and berries						
Cases/follow-up	158/19.4	102/19.8	63/20.0	58/20.4	48/20.4	
Mean intake (g/1000 kcal)	23	53	79	112	187	
Basic model^a^	1.00	0.66 (0.52*–*0.86)	0.45 (0.34*–*0.61)	0.44 (0.32*–*0.60	0.42 (0.30*–*0.59)	0.78 (0.72*–*0.84)
Multivariable model^b^	1.00	0.82 (0.63*–*1.05)	0.64 (0.47*–*0.87)	0.67 (0.48*–*0.92)	0.67 (0.47*–*0.95)	0.89 (0.82*–*0.96)
Excluding misreporters^c^	1.00	0.76 (0.56*–*1.05)	0.70 (0.49*–*1.01)	0.76 (0.51*–*1.11)	0.60 (0.37*–*0.97)	0.89 (0.81*–*0.97)
Fruits, citrus						
Cases/follow-up	141/19.3	98/19.9	76/20.1	62/20.3	52/20.4	
Mean intake (g/1000 kcal)	1	5	11	19	44	
Basic model^a^	1.00	0.74 (0.57*–*0.95)	0.62 (0.47*–*0.82)	0.55 (0.41*–*0.74)	0.50 (0.36*–*0.70)	0.83 (0.78*–*0.90)
Multivariable model^b^	1.00	0.86 (0.66*–*1.12)	0.78 (0.59*–*1.04)	0.75 (0.55*–*1.01)	0.68 (0.49*–*0.95)	0.91 (0.85*–*0.98)
Excluding misreporters^c^	1.00	0.99 (0.72*–*1.36)	0.76 (0.53*–*1.09)	0.84 (0.58*–*1.22)	0.70 (0.45*–*1.08)	0.92 (0.83*–*1.00)
Fruits, non-citrus						
Cases/follow-up	165/19.2	92/19.9	67/20.1	56/20.5	49/20.4	
Mean intake (g/1000 kcal)	14	36	56	83	144	
Basic model^a^	1.00	0.58 (0.45*–*0.75)	0.46 (0.35*–*0.62)	0.42 (0.31*–*0.57)	0.41 (0.30*–*0.58)	0.80 (0.72*–*0.84)
Multivariable model^b^	1.00	0.74 (0.57*–*0.96)	0.62 (0.46*–*0.83)	0.61 (0.44*–*0.84)	0.65 (0.46*–*0.91)	0.87 (0.81*–*0.95)
Excluding misreporters^c^	1.00	0.61 (0.44*–*0.85)	0.59 (0.41*–*0.85)	0.61 (0.41*–*0.90)	0.62 (0.39*–*0.98)	0.87 (0.78*–*0.96)

^a^Adjusted for age, sex, season, method and total energy intake

^b^Adjusted for age, sex, season, method, total energy intake, physical activity, education, alcohol, smoking and BMI

^c^Adjusted for age, sex, season, method, total energy intake, physical activity, education, alcohol, smoking, BMI and excluded dietary changers and misreporters

Analyses were also carried out on the individual food groups for refined grains and whole grains, i.e. soft bread, crisp bread and cereals. However, no statistically significant associations were identified for these variables and risk of developing AAA in the multivariable model (supplementary Table 3). Analyses were also carried out on juice, but no significant associations were found. To test the role of hypertension, this variable was added to the multivariable model, and the results remained essentially the same.

Due to the risk of residual confounding with smoking status, we also run the analyses adjusting for cigarettes per day instead of smoking status in the multivariable model; however, the associations were virtually unchanged. We also stratified the analyses for smoking status. When only including current smokers, the associations were weaker for total vegetables (HR 0.87, 95% CI 0.78–0.96) and leaf vegetables (HR 0.82, 95% CI 0.74–0.91) and remained essentially the same for fruits and berries (HR 0.90, 95% CI 0.81–1.00), citrus fruits (HR 0.92, 95% CI 0.84–1.01), non-citrus fruits (HR 0.90, 95% CI 0.81–1.00) and fibre (HR 0.91, 95% CI 0.83–1.01). When only including former smokers and non-smokers, no associations were found for total vegetables (HR 0.98, 95% CI 0.87–1.10), leaf vegetables (HR 0.93, 95% CI 0.83–1.05), citrus fruits (HR 0.90, 95% CI 0.80–1.01) and fibre (HR 0.95, 95% CI 0.84–1.06), and the risk was principally unchanged for fruits and berries (HR 0.87, 95% CI 0.77–0.99) and non-citrus fruits (HR 0.84, 95% CI 0.75–0.95). However, there were no significant interaction between smoking status and leaf vegetables (*p* = 0.19), total vegetables (*p* = 0.29), fruits and berries (*p* = 0.31), fibre (*p* = 0.94), citrus fruits (*p* = 0.43) and non-citrus fruits (*p* = 0.17) in AAA risk.

## Discussion

This study shows a lower risk of developing AAA with higher intake of plant foods, such as fruits and vegetables. These associations were upheld after adjusting for several confounders.

The strongest association in this study was identified for total vegetable intake and reduced AAA risk, as was shown in the sensitivity analysis where vegetable consumers in the fifth quintile had a 43% lower risk compared to the first quintile. A similar result was identified for intake of fruits and berries, where the consumers in the fifth quintile had a 40% reduced risk of AAA, when compared to the lowest quintile. These findings are in line with previous research conducted in the same cohort, with a shorter follow-up period [12]. Another previous cohort study investigating risk of AAA in association with fruits and vegetables consumption found that the highest quartile of fruit consumers had a reduced risk of AAA compared to the lowest quartile, an association that could not be observed for vegetable consumption [13]. Differences in dietary assessment method and shorter follow-up period might explain the deviations in results regarding vegetable consumption and AAA risk, compared to the present study.

This study was also able to ascertain associations between intake of whole grains and reduced risk of AAA after excluding potential misreporters and individuals with unstable food habits. Whole grains include all cereal food products with a high fibre content, such as high-fibre bread and cereals. AAA and CVD have a similar aetiology with numerous risk factors in common [6, 24]. An association between a high intake of whole grains and decreased risk of incident CVD, defined as myocardial infarction and stroke, has previously been identified in the MDCS [25]. The highest quintile had a 19% lower risk of incident CVD compared to the lowest quintile. These findings are in line with the results of the present study where the highest intake of whole grains was associated with a 36% reduced risk of AAA. Additionally, these results are in accordance with the findings of a cohort study in the US where an association between adhering to the Dietary Approaches to Stop Hypertension (DASH) dietary pattern and reduced AAA risk was established [26]. A high intake of whole grain is a component of the DASH diet and the study concluded that the highest quintile of whole grain consumers had a 33% lowered AAA risk, when compared to the lowest quintile [26]. However, the present study is believed to be the first to establish an association between whole grain consumption and AAA risk, outside of the scope of a lifestyle or diet intervention.

The results from the analyses of subgroups of fruits and vegetables showed that high intake of leaf vegetables was associated with reduced AAA risk. No previous research investigating leaf vegetables and AAA risk has been identified. However, it has been suggested that inorganic nitrate, which is common in green leafy vegetables, is protective against cardiovascular risk factors such as high blood pressure and arterial stiffness [27], which might offer some explanation to the finding in the present study. Another finding is that the risk of AAA was similar for non-citrus and citrus fruits. This suggests that fruit is protective against AAA, and that the protective component in fruits is not citrus but rather something else. One plausible explanation to these findings are that fruits and vegetables are rich in antioxidants, such as quercetin, polyphenol and vitamin E, believed to reduce oxidative stress in the aortic wall and thus act as a potential inhibitor to AAA growth [28]. Moreover, fruits and vegetables are often anti-inflammatory and it has been suggested that inflammation plays a key role, not only in the clinical presence of AAA, but also in relation to the pathogenesis. Increased C-reactive protein (CRP) levels and higher concentrations of inflammatory cells, such as macrophages and lymphocytes, in the aorta are examples of the association between AAA and inflammation [28].

Since smoking is a major risk factor for AAA, separate analyses were conducted on smokers and non-smokers. The results showed that for smokers, intake of vegetables, and in particular leaf vegetables, is associated with reduced AAA risk. For non-smokers, an association between fruit intake and reduced AAA risk was established, while no association was identified for vegetables. A recent systematic review found that smoke, nicotine or tobacco products increased aortic dimension and induced metalloproteinase (MMP), MMP-1, MMP-2, MMP-8, MMP-9 and MMP-12, through various molecular mechanisms such as JNK, AMPK-a2, Jak Stat, and mTOR/p70Sk and PTEN pathways. It may be that vegetable and fruit constituents interrupt some of these molecular pathways leading to altering of MMPs and the formation of AAA [29].

Given that the pathogenesis of AAA is not yet completely known, it remains challenging to completely elucidate why a high intake of fibre is associated with a reduced AAA risk in this cohort. It is plausible that those who consume higher levels of fibre also have a healthier lifestyle in general and are thus healthier. This has been illustrated in a study which concluded that a low-risk lifestyle was associated with decreased risk of diverticulitis. A low-risk lifestyle incorporated, in this study, for instance a high intake of fibre, vigorous physical activity and maintaining a normal BMI [30]. The present study identified that there appears to be associations between physical activity and education levels and reduced AAA risk. This warrants further investigation in future studies as it can help to further illuminate the relationship between maintaining a healthy lifestyle and reduced risk of developing AAA.

A major strength of the present study is the large study sample comprising of over 30,000 individuals as well as the long follow-up time of approximately 20 years. Since AAA risk generally increases with age, it is highly suitable for the purposes of this study to have an elderly study population. Another strength of this cohort study was the high validity, 95%, of AAA diagnosis. It is well known that physicians have difficulties in distinguishing aortic aneurysm from aortic dissection, and subgroup classification of aortic aneurysm in administrative databases are inexact, with large group of aortic aneurysm of unspecific site [19], resulting in erroneous coding of aortic diseases [31]. In addition, using registries to attain disease endpoints is another significant strength as it ensured almost complete follow-up of the study participants. The MDCS was also designed with the intention to measure intake of fibre and the fibre content of for instance bread was carefully examined at baseline. Finally, the study design used at baseline data collection has been validated and showed lower levels of misreporting than other, similar methodologies [32].

Potential weaknesses to this study is the self-administered structure of the majority of baseline dietary data collection and the relatively low participation rate of 40%, which might affect the external validity, [20]. Moreover, the overrepresentation of women in the MDCS is a potential issue when investigating AAA, a condition primarily affecting men. In addition, no ultrasound measurements of the aortic diameter were conducted at baseline to ensure that the study participants did not have asymptomatic, small AAA. Hence, it is possible that a few individuals with small AAA were erroneously included in the present study. It is also probable that some individuals changed their dietary habits during the follow-up period and, therefore, do not have the same risk as they did at baseline. However, a longitudinal study following middle-aged individuals attempting fat reduction showed that after 24 months, almost 70% had not altered their diet [33]. Regardless, those who stated that they had changed their diet in the past, noted as dietary changers in the statistical analysis, were omitted in the ‘excluding misreporters’ model, to conclude whether these individuals impacted the results in any way.

In conclusion, an association between intake of fibre and reduced AAA risk was found. These findings support the growing evidence outlining the health benefits, and the reduced risk of certain diseases, associated with maintaining a diet rich in fibre.

## Electronic supplementary material

Below is the link to the electronic supplementary material.

Supplementary material 1 (DOCX 17 kb)
