# Supplementary material for: Intake of fibre and plant foods and the risk of abdominal aortic aneurysm in a large prospective cohort study in Sweden
Source: Eur J Nutr. 2019 Jul 22;59(5):2047–56. doi: 10.1007/s00394-019-02054-w (PMC7351812; doi:10.1007/s00394-019-02054-w)
Supplement: Supplementary file 1 — Supplementary material 1 (DOCX 17 kb) [file 394_2019_2054_MOESM1_ESM.docx]

Supplementary table 3. HR and 95% CI for energy adjusted food groups, divided according to categories of intake among participants in the Malmö Diet and Cancer Cohort.

|  | Intake categories | | | | |  |
| --- | --- | --- | --- | --- | --- | --- |
|  | 1 | 2 | 3 | 4 | 5 | Per intake category |
| **Low-fibre soft bread** |  |  |  |  |  |  |
| Cases/follow-up | 67/20.4 | 72/20.1 | 70/20.00 | 110/19.9 | 110/19.7 |  |
| Mean intake (g/1000 kcal) | 8 | 21 | 32 | 45 | 72 |  |
| Basic model^a^ | 1.00 | 1.07(0.76-1.49) | 0.95 (0.68-1.34) | 1.35 (0.996-1.84) | 1.21 (0.89-1.66) | 1.07 (0.995-1.14) |
| Multivariable model^b^ | 1.00 | 1.06 (0.76-1.48) | 0.95 (0.67-1.33) | 1.28 (0.93-1.75) | 1.17 (0.85-1.61) | 1.05 (0.98-1.13) |
| Excluding misreporters^c^ | 1.00 | 1.23 (0.79-1.92) | 1.16 (0.75-1.80) | 1.33 (0.87-2.02) | 1.37 (0.90-2.08 | 1.07 (0.98-1.17) |
| **Low-fibre crisp bread** |  |  |  |  |  |  |
| Cases/follow-up | 63/20.4 | 102/20.1 | 92/19.9 | 88/19.8 | 84/20.0 |  |
| Mean intake (g/1000 kcal) | 0 | 0 | 1 | 1 | 5 |  |
| Basic model^a^ | 1.00 | 1.50 (1.10-2.06) | 1.36 (0.99-1.87) | 1.30 (0.94-1.80) | 1.47 (1.06-2.05) | 1.06 (0.99-1.13) |
| Multivariable model^b^ | 1.00 | 1.45 (1.06-1.99) | 1.27 (0.92-1.76) | 1.17 (0.84-1.62) | 1.42 (1.02-1.97) | 1.04 (0.97-1.12) |
| Excluding misreporters^c^ | 1.00 | 1.39 (0.94-2.06) | 1.22 (0.82-1.82) | 1.13 (0.76-1.69) | 1.39 (0.92-2.09) | 1.04 (0.95-1.13) |
| **Cereals wholegrain** |  |  |  |  |  |  |
| Cases/follow-up | 259/19.8 | 56/20.7 | 51/20.1 | 63/19.9 |  |  |
| Mean intake (g/1000 kcal) | 0 | 0 | 2 | 13 |  |  |
| Basic model^a^ | 1.00 | 0.78 (0.58-1.04) | 0.64 (0.47-0.87) | 0.70 (0.53-0.92) |  | 0.86 (0.79-0.94) |
| Multivariable model^b^ | 1.00 | 0.92 (0.68-1.24) | 0.74 (0.55-1.01) | 0.85 (0.64-1.13) |  | 0.92 (0.85-1.01) |
| Excluding misreporters^c^ | 1.00 | 0.78 (0.53-1.14) | 0.81 (0.57-1.16) | 0.80 (0.55-1.14) |  | 0.92 (0.82-1.02) |
| **Cereals refined** |  |  |  |  |  |  |
| Cases/follow-up | 272/19.8 | 48/20.3 | 53/20.2 | 56/20.4 |  |  |
| Mean intake (g/1000 kcal) | 0 | 2 | 5 | 12 |  |  |
| Basic model^a^ | 1.00 | 0.74 (0.55-1.01) | 0.75 (0.56-1.00) | 0.72 (0.54-0.95) |  | 0.88 (0.81-0.96) |
| Multivariable model^b^ | 1.00 | 0.85 (0.62-1.16) | 0.89 (0.66-1.20) | 0.89 (0.66-1.19) |  | 0.95 (0.87-1.04) |
| Excluding misreporters^c^ | 1.00 | 0.86 (0.59-1.27) | 1.02 (0.72-1.45) | 0.79 (0.54-1.16) |  | 0.95 (0.85-1.07) |
| **Rice/pasta** |  |  |  |  |  |  |
| Cases/follow-up | 161/19.40 | 107/20.10 | 95/20.18 | 66/20.48 |  |  |
| Mean intake (g/1000 kcal) | 0 | 3 | 6 | 14 |  |  |
| Basic model^a^ | 1.00 | 0.92 (0.72-1.17) | 0.87 (0.68-1.13) | 0.63 (0.47-0.84) |  | 0.88 (0.81-0.96) |
| Multivariable model^b^ | 1.00 | 0.94 (0.73-1.21) | 0.98 (0.76-1.27) | 0.73 (0.54-0.98) |  | 0.93 (0.85-1.01) |
| Excluding misreporters^c^ | 1.00 | 0.99 (0.73-1.34) | 1.02 (0.74-1.40) | 0.83 (0.58-1.19) |  | 0.96 (0.86-1.07) |
| **Biscuits and rusks** |  |  |  |  |  |  |
| Cases/follow-up | 310/19.9 | 33/20.6 | 38/20.3 | 48/19.7 |  |  |
| Mean intake (g/1000 kcal) | 0 | 0 | 1 | 5 |  |  |
| Basic model^a^ | 1.00 | 0.63 (0.44-0.91 | 0.74 (0.53-1.04) | 0.96 (0.70-1.30) |  | 0.94 (0.85-1.03) |
| Multivariable model^b^ | 1.00 | 0.75 (0.52-1.08) | 0.84 (0.60-1.18) | 1.02 (0.74-1.39) |  | 0.97 (0.88-1.07) |
| Excluding misreporters^c^ | 1.00 | 0.63 (0.39-1.02) | 0.83 (0.55-1.26) | 0.92 (0.62-1.37) |  | 0.94 (0.84-1.06) |
| **High-fibre soft bread** |  |  |  |  |  |  |
| Cases/follow-up | 247/19.5 | 57/20.4 | 55/20.6 | 70/20.5 |  |  |
| Mean intake (g/1000 kcal) | 0 | 3 | 11 | 34 |  |  |
| Basic model^a^ | 1.00 | 0.74 (0.56-00) | 0.73 (0.54-0.98) | 0.81 (0.62-1.06) |  | 0.91 (0.84-0.99) |
| Multivariable model^b^ | 1.00 | 0.87 (0.65-1.16) | 0.88 (0.65-1.18) | 0.96 (0.73-1.26) |  | 0.97 (0.89-1.06) |
| Excluding misreporters^c^ | 1.00 | 0.93 (0.66-1.32) | 0.89 (0.62-1.29) | 0.81 (0.56-1.17) |  | 0.94 (0.84-1.04) |
| **High-fibre crisp bread** |  |  |  |  |  |  |
| Cases/follow-up | 155/19.2 | 93/20.2 | 101/20.2 | 80/20.4 |  |  |
| Mean intake (g/1000 kcal) | 0 | 2 | 5 | 15 |  |  |
| Basic model^a^ | 1.00 | 0.62 (0.48-9.80) | 0.70 (0.54-0.90) | 0.60 (0.46-0.79) |  | 0.85 (0.78-0.93) |
| Multivariable model^b^ | 1.00 | 0.72 (0.55-0.94) | 0.87 (0.68-1.13) | 0.74 (0.56-0.97) |  | 0.92 (0.84-1.01) |
| Excluding misreporters^c^ | 1.00 | 0.75 (0.54-1.03) | 0.84 (0.61-1.16) | 0.78 (0.55-1.10) |  | 0.93 (0.83-1.04) |

^a^ adjusted for age, sex, season, method and total energy intake

^b^ adjusted for age, sex, season, method, total energy intake, physical activity, education, alcohol, smoking and BMI

^c^ adjusted for age, sex, season, method, total energy intake, physical activity, education, alcohol, smoking, BMI and excluded dietary changers and misreporters

Supplementary Table 4. Validation of patients with AAA and rAAA

| **Characteristics** | **AAA (n=80)** | **rAAA (n=18)** | **P-value** |
| --- | --- | --- | --- |
| Confirmed diagnosis (%) | 76/80 (95%) | 17/18 (94.4) | 1.0 |
| Median (IQR) age; years | 76 (71 – 80) | 84 (78 – 85) | <0.001 |
| Male sex, (%) | 58/76 (76.3) | 12/17 (70.6) | 0.62 |
| Median maximal AAA diameter (IQR); mm | 50 (40 – 64) | 78 (56 – 98) | <0.001 |
| Operated at the time of diagnosis, (%)  EVAR  Open repair | 5/76 (6.6)  5  0 | 10/17 (58.8)  8  2 | <0.001 |
| Mortality within 30 days of diagnosis, (%) | 0/76 (0) | 11/17 (64.7) | <0.001 |
| Initial mode of diagnosis, (%)  Imaging due to AAA-related symptoms  Accidental finding at any imaging  Palpation  Autopsy  Organized ultrasound screening for AAA  Non-organized ultrasound screening for AAA | 2  56  12  0  2  4 | 16  0  0  1  0  0 |  |

AAA; abdominal aortic aneurysm, rAAA; ruptured abdominal aortic aneurysm, EVAR; Endovascular aneurysm repair, IQR; interquartile range
